# Enhanced Glycosylation Caused by Overexpression of Rv1002c in a Recombinant BCG Promotes Immune Response and Protects against *Mycobacterium tuberculosis* Infection

**DOI:** 10.3390/vaccines12060622

**Published:** 2024-06-04

**Authors:** Shufeng Weng, Qingchun Li, Tianran Zhang, Taiyue Lin, Yumo He, Guang Yang, Honghai Wang, Ying Xu

**Affiliations:** 1 Department of Infectious Diseases, National Medical Center for Infectious Diseases, State Key Laboratory of Genetic Engineering, Shanghai Key Laboratory of Infectious Diseases and Biosafety Emergency Response, School of Life Sciences, Huashan Hospital, Fudan University, Shanghai 200437, China; 20110700013@fudan.edu.cn (S.W.); 23210700133@m.fudan.edu.cn (Q.L.); 18210700051@fudan.edu.cn (T.Z.); 22210700032@m.fudan.edu.cn (T.L.); 22210700114@m.fudan.edu.cn (Y.H.); 23210700069@m.fudan.edu.cn (G.Y.); hhwang@fudan.edu.cn (H.W.); 2 Shanghai Sci-Tech Inno Center for Infection & Immunity, Shanghai 200052, China

**Keywords:** *Mycobacterium tuberculosis*, vaccine, rBCG-Rv1002c, glycoprotein, immune protection

## Abstract

Tuberculosis (TB) is a major global health threat despite its virtual elimination in developed countries. Issues such as drug accessibility, emergence of multidrug-resistant strains, and limitations of the current BCG vaccine highlight the urgent need for more effective TB control measures. This study constructed BCG strains overexpressing Rv1002c and found that the rBCG-Rv1002c strain secreted more glycosylated proteins, significantly enhancing macrophage activation and immune protection against *Mycobacterium tuberculosis* (*M. tb*). These results indicate that Rv1002c overexpression promotes elevated levels of O-glycosylation in BCG bacteriophages, enhancing their phagocytic and antigenic presentation functions. Moreover, rBCG-Rv1002c significantly upregulated immune regulatory molecules on the macrophage surface, activated the NF-κB pathway, and facilitated the release of large amounts of NO and H_2_O_2_, thereby enhancing bacterial control. In mice, rBCG-Rv1002c immunization induced greater innate and adaptive immune responses, including increased production of multifunctional and long-term memory T cells. Furthermore, rBCG-Rv1002c-immunized mice exhibited reduced lung bacterial load and histological damage upon *M. tb* infection. This result shows that it has the potential to be an excellent candidate for a preventive vaccine against TB.

## 1. Introduction

Tuberculosis (TB) is a major infectious disease that is a serious threat to human health. Despite being virtually eliminated in rich countries, it remains the single largest cause of death from a single disease worldwide [1]. Notwithstanding the existence of recommended antibiotics for TB treatment, issues of drug accessibility, the rise of multidrug-resistant TB and extensively multidrug-resistant TB (XMDR-TB) [2], and other factors that contribute to TB remaining a global public health problem. According to the World Health Organization (WHO), there were approximately 10 million new TB cases and up to 1.3 million deaths worldwide in 2022 [3]. Since 1921, BCG, an inhibitory strain of *Mycobacterium bovis* (*M. bovis*), has been the only TB vaccine licensed for clinical use, protecting against disseminated TB in infants but with poor protection in adolescents and adults [4]. Therefore, there is an urgent need for a more effective vaccine to control TB.

Currently, 13 vaccine candidates are in clinical trials, and all booster vaccines follow BCG immunization, except for two recombinant vaccines (VPM1002 and MTBVAC) used to replace BCG [5]. The main goal of preventive vaccine research is to create vaccines that are more effective than BCG. One of them is VPM1002, a recombinant BCG vaccine that replaces the urease C gene in BCG with the gene-encoding *Listeria monocytogenes* hemolysin (*Hly*), which can induce a strong Th1 response [6] or produce completely new vaccines, such as the rhesus cytomegalovirus tuberculosis vaccine (RhCMV/TB), including vectors expressing nine different *Mycobacterium tuberculosis* (*M. tb*) proteins that activate and maintain a high-frequency T cell response and protect against *Mycobacterium tuberculosis* [7]. Another option is the use of alternative routes of vaccination, such as oral BCG [8], which is effective in activating mucosal immunity [9], and intravenous administration [10]; however, its safety has been questioned, and it is the most potent of all routes of immunity [11]. ID93, a vaccine that can stimulate CD4^+^ T cells to develop into multifunctional CD4^+^ T cells [12,13], and H56: IC31, a subunit vaccine that stimulates the host to produce high levels of IL-2 and TNF-α [14,15], are two primary immunization-boosting approaches to enhancing BCG protection.

It has recently been reported that *M. tb* glycoproteins are also a class of bacterial adhesins that can promote *M. tb* infectivity by binding to host immune receptors [16,17], including lung surface-active protein A (SP-A) [18] and DC-sign [19], which are thought to be receptors preferentially used by *Mycobacteria* for entry into the target cells and to evade host defense mechanisms [20]. *M. tb* glycoproteins can modulate host immunity, e.g., alterations in the mannosylation pattern of Apa proteins alter their ability to stimulate CD4^+^ and CD8^+^ T lymphocytes [21,22], and the glycosylation of the lipoprotein LprG is critical for MHC II-restricted T cell activation in leprosy patients [23,24,25]. Rv1002c is the only O-mannosyltransferase in *M. tb* that catalyzes the transfer of mannose to the Ser or Thr residues of the peptide chain [26,27]. Most O-mannosylated proteins are secreted, and bacterial surface proteins in *M. tb* are related to virulence [27,28].

In this study, we constructed BCG strains overexpressing Rv1002c. We found that the rBCG-Rv1002c strain secreted more glycosylated proteins than BCG, significantly promoting macrophage activation, secreting a large amount of NO and H_2_O_2_ after the immunization of mice, and further promoting T cell activation and proliferation, with a significant increase in the proportion of multifunctional T cells, presenting stronger immune protection against *M. tb*. These results suggest that rBCG-Rv1002c is a potential vaccine candidate and may replace BCG in immunoprotecting against TB.

## 2. Materials and Methods

### 2.1. Ethics Statement

All mice were maintained under the specific pathogen-free conditions at the Animal Center of the School of Life Sciences of Fudan University. All experimental procedures were approved by the Animal Care and Use Ethics Committee of Fudan University under license number 2019JS016. Following the Fudan University biosafety rules, BCG vaccination experiments were conducted in the ABSL-2 laboratory, and *M. tb*-related investigations were conducted in the ABSL-3 laboratory.

### 2.2. Bacteria and Cell Lines

The method of enzymatic ligation was used to construct pMV261-*rv1002c* and pET28a-*rv1002c*. Sequences encoding Rv1002c were amplified from *M. tb* H37Rv by PCR using five pairs of primers (F: aaGGATCCgtggtacccgtcgtcagccccg, R: ttAAGCTTccgccagctgggcagccagatct; Sangon Biotech Co., Ltd., Shanghai, China). Recombinant plasmid was constructed by ligating the amplified fragment digested with restriction endonucleases (BamHI, HindIII) to the linearized plasmid. DNA was sequenced by Shanghai Sangon and was found to conform to the designed sequences using BLAST analysis. The successfully constructed plasmid was electrotransformed into the BCG receptor strain, and the empty vector pMV261 was also electrotransformed to construct the BCG-Vec strain. The pET28a-*rv1002c* was transformed into the *Escherichia coli* BL21 strain and validated, followed by IPTG-induced affinity purification of the Rv1002c protein by Ni-nitrilotriacetic acid. The *Escherichia coli* DH5α strain was cultured in LB medium to clone and purify the plasmids, and the BL21 strain was used for protein purification. The rBGC-Rv1002c overexpressing Rv1002c was constructed using the BCG-Danish strain. *M. tb* H37Rv and BCG cultures were grown in 7H9 broth (Cat#271310; Difco, Tucker, GA, USA) and cultures were grown to logarithmic growth. Jurkat and THP-1 cells were cultured in RPMI 1640 + 10% FBS + 1% PS (Gibco, Big Cabin, OK, USA).

### 2.3. Mouse Immunizations and M. tb Infections

BCG cultured to the log phase was collected by centrifugation, washed twice with PBS, resuspended, and adjusted to an OD600 value of 1.0. The 6-week-old C57BL/6j mice received immunizations of 100 μL of OD600 = 1.0 bacterial culture (2 × 10^7^ CFU/mL) in PBS subcutaneously in the left flank of either BCG or rBCG-Rv1002c. Some mice received H37Rv prepared similarly for immunoprotective studies. Mice rested four weeks post-vaccination before intranasal challenge with 300 CFU of *M. tb* H37Rv. Bacterial burden was estimated by plating serial dilutions of lung and spleen homogenates on 7H10 (Cat#262710; Difco, USA) plates, as indicated. Selected lung tissues were fixed in 4% paraformaldehyde and stained by H&E for histological assessment. The lungs were assessed pathologically using a four-level grading system.

### 2.4. Phagocytosis Assay of Macrophages

THP-1 cells were cultured in 24-well plates and treated with 100 ng/mL PMA (Cat#P8139; Sigma, St. Louis, MO, USA) for 48 h, infected with BCG, BCG-Vec, and rBCG-Rv1002c at a 10:1 ratio for 3 h, and cultured for 12 h. Subsequently, heat-inactivated *M. tb-mCherry* with five-fold THP-1 was added. The medium was replaced with fresh medium, and the cells were washed three times with PBS after 12 h of incubation, stained with DAPI (Cat#C1002; Beyotime, China), and observed using a confocal microscope (Olympus FV3000, Tokyo, Japan).

For co-localization experiments, cells were infected with *Mycobacterium* at the indicated MOI for 4 h. After removal of extracellular bacteria by washing with PBS, the cells were fixed with 4% paraformaldehyde at different time intervals. The fixed cells were permeabilized with 0.1% Triton X-100 and then immunofluorescent-labelled according to standard procedures. Rabbit Abs (Cat#ab214721; Abcam, Waltham, MA, USA) for BCG, mouse Abs (Cat#AG4368; Beyotime, Nantong, China) for p-P65, and mouse Abs (Cat#AG2741; Beyotime, China) for IκBα were used. Secondary Abs, anti-rabbit IgG-Alexa555, and anti-mouse IgG-Alexa488 were used for staining. Cells were then examined by laser scanning microscopy using a confocal microscope (Olympus FV3000, Japan).

### 2.5. Isolation of RNA and RT-qPCR

After washing the infected THP-1 cells three times with PBS, total RNA was extracted using TRIzol (Cat#15596026; Invitrogen, Waltham, MA, USA). Briefly, 1 µg of isolated RNA was immediately reverse-transcribed into cDNA using a HiScript III 1st Strand cDNA Synthesis Kit (Cat#R312-01; Vazyme, Nanjing, China). The Taq Pro Universal SYBR qPCR Master Mix (Cat#Q712-02; Vazyme, China) was used for RT-qPCR analysis of myD88, TAK1, TRIFR, and TRAMR expression. Data from three independent experiments were used for statistical analyses.

### 2.6. Western Blotting

WCL containing 15 μg of total protein was separated using SDS-PAGE (10% acrylamide gels) and transferred onto a PVDF membrane (Cat# IPVH00010; Millipore, Burlington, MA, USA). After blocking with 5% skim milk for 2 h, the membrane was incubated with p-P65 (Cat#AF5875; Beyotime, China), P65 (Cat#AF0246; Beyotime, China), IκBα (Cat#AF1282; Beyotime, China), O-GlcNAC (Cat#PTM952; PTM BIO, Hangzhou, China), or Biotin-ConA (Cat#C2272; Sigma, USA) antibodies. After washing, HRP-conjugated goat anti-rabbit IgG antibody (Cat#SA00001-2; Proteintech, Wuhan, China) or HRP-conjugated goat anti-mouse IgG antibody (Cat#SA00001-1; Proteintech, China) was added as a secondary antibody. After washing, bands were developed using an ultrasensitive ECL substrate (Cat#SQ201; Epizyme, Shanghai, China).

### 2.7. Mixed Lymphocyte Cultivate (MLC)

THP-1 cells were cultured in 24-well plates and treated with 100 ng/mL PMA (Cat#P8139; Sigma, USA) for 48 h, infected with BCG, BCG-Vec, and rBCG-Rv1002c at a 5:1 ratio for 3 h, and then cultured for 12 h. Subsequently, Jurkat cells were added 10 times the amount of THP-1 and replaced with fresh culture medium. The culture was continued for 72 h before cells were collected for flow cytometry.

### 2.8. Functional Factor Assay and ELISA

Peritoneal cells were collected from immunized mice, 3 × 10^5^ cells were added to a U-bottom 96-well culture plate, and 5 μg/mL Rv1002c was added to stimulate cells for 12 h. Cell supernatants were collected for NO (Cat#AKNM005C; Boxbio, Shanghai, China), H_2_O_2_ (Cat#AKAO009C; Boxbio, China), TNF-α (Cat#1217202; Dakewe, Shenzhen, China), and IFN-γ (Cat# 1210012; Dakewe, China) assays. The procedure was performed strictly according to the manufacturer’s instructions.

### 2.9. Flow Cytometry

Live cells were discriminated against using a live/dead cell stain (Cat# 423107; BioLegend, San Diego, CA, USA). Splenocytes (2 × 10^6^ cells/well) were cultured in 96-well U-bottom plates with RPMI + 10% FBS for 16 h, with or without Rv1002c protein (5 µg/mL). Brefeldin A (BFA, final concentration: 5 µg/mL, Cat# HY-16592; MCE, Foshan, China) was added in 5 h before staining. After PBS washing, anti-CD3ε PerCP-Cy5.5 (Cat#155615; BioLegend, USA), anti-CD4 BV510 (Cat#100559; BioLegend, USA), anti-CD8a FITC (Cat#100705; BioLegend, USA), anti-CD69 PE-Cy7 (Cat#104511; BioLegend, USA), anti-CD44 BV605 (Cat#103047; BioLegend, USA), and anti-CD62L PE-Cy7 (Cat#104417; BioLegend, USA) were used to stain surface markers. Then, anti-CD107a AF700 (Cat#121627; BioLegend, USA), anti-IL-2 APC (Cat#503809; BioLegend, USA), anti-IFN-γ BV605 (Cat#505840; BioLegend, USA), anti-IL-17A PE (Cat#506903; BioLegend, USA), and anti-TNF-α BV421 (Cat#506327; BioLegend, USA) were stained intracellularly with Cyto-Fast™ Fix/Perm (Cat#426803; BioLegend, USA) according to the manufacturer’s instructions. FMO controls and isotype controls were applied to aid in data processing.

Data were immediately acquired using the BD Fortessa flow cytometer (BD Biosciences, Lakes, NJ, USA). The data were analyzed using FlowJo V10 software (BD Biosciences).

### 2.10. Mass Spectrometry Analysis

Bacteriophage supernatant proteins were concentrated with ammonium sulphate and quantified by the BCA method, and 100 ug of protein was taken for mass spectrometry.

After three displacements with 8 M urea (Cat#U111898; Aladdin, Shanghai, China) and 100 mM Tris-HCl (pH 8.5) buffer, the proteins were reduced with 10 mM DTT (Cat#646563; Sigma Aldrich, St. Louis, MO, USA) for 30 min at 37 °C, followed by alkylation with 30 mM iodoacetamide for 25 °C in the dark for 45 min. Digestion with trypsin (enzyme/protein 1:50) (Cat#T9201; Sigma Aldrich, USA) was carried out at 37 °C for 12 h. After digestion, the filtrate was rinsed twice with 15% ACN, then all filtrates were pooled and dried under vacuum to a final concentration of 1 mg/mL. LC-MS analyses were performed using a nanoflow EASYnLC 1200 system (Thermo Fisher Scientific, Odense, Denmark) coupled to an Orbitrap Fusion Lumos mass spectrometer (Thermo Fisher Scientific, Bremen, Germany). Samples were analyzed on a homemade C18 automated optical column (inner diameter 75 µm × 25 cm, ReproSil-Pur 120 C18-AQ, 1.9 µm; Dr. Maisch GmbH, Ammerbuch, Germany). Derived peptides were eluted with the following gradient, 2–5% B for 2 min, 5–35% B for 100 min, 35–44% B for 6 min, 44–100% B for 3 min, and 100% B for 10 min, at a flow rate of 200 nL/min.

### 2.11. RNA-Seq

Total RNA was extracted from mouse lungs. The mRNA was fragmented into short fragments of approximately 200–500 nucleotides (nt) using Fragmentase, and then the fragments were used as templates for the synthesis of the first strand of cDNA, with dUTP replacing dTTP in the synthesis of the second strand. After purification of the short fragments, end repair and single nucleotide A (adenine) addition was performed with elution buffer. Subsequently, the short fragment and second strand associated with the aptamer were degraded using UNG. After agarose gel electrophoresis, suitable fragments were selected as templates for PCR amplification and quality control. Finally, Illumina HiSeq4000 sequenced the sample library using the PE100 strategy. The resulting raw sequencing data, called raw reads, were converted to clean reads by removing low-quality reads with BGI’s software (https://bgi.obs-mip.fr, accessed on 10 May 2023). Data analysis was performed using RStudio (https://www.rstudio.com/, accessed on 12 May 2023). Potentially expressed genes were identified by linear modal analysis and Bayesian statistics using the Limma software package (https://bioconductor.org/packages/release/bioc/html/limma.html, accessed on 12 May 2023). Pathway analysis was performed using gene set enrichment analysis (GSEA; http://software.broadinstitute.org/gsea/index.jsp, accessed on 12 May 2023).

### 2.12. Mouse Peritoneal Cell Isolation

After the immunized mice were killed, 5 mL of cell culture medium was aspirated with a syringe and slowly injected into the abdominal cavity of the mice, and then the abdomen of the mice was gently kneaded several times and left for 3–5 min to allow the liquid to flow sufficiently into the abdominal cavity. The abdominal skin was separated from the peritoneum with forceps and the entire abdominal skin was cut through to expose the peritoneum. The viscera were squeezed by manually pinching the skin on one side of the mouse to create a cavity in the abdominal cavity, extracting the peritoneal fluid, and centrifuging to collect the cells for flow staining.

### 2.13. Mouse Splenic Single Cell Isolation

After the immunized mice were killed, the spleens of mice were removed and placed in folded sterile gauze, and the spleens were ground in a Petri dish using 1640 medium containing 10% FBS to obtain a single cell suspension. The ground cell suspension was centrifuged at 1000 rpm for 5 min. The supernatant was discarded and the erythrocytes were lysed by the addition of erythrocyte lysate, and then lysis was terminated by the addition of FBS-containing 1640 medium and collected by centrifugation again for flow staining.

### 2.14. Statistical Analysis

Statistical analyses were performed using GraphPad Prism 8 (GraphPad Software Inc., La Jolla, CA, USA). Comparisons between two groups were conducted using *t*-tests. Comparisons between three or more groups were performed using one-way ANOVA. A *p* < 0.05 was considered to indicate statistical significance.

## 3. Results

### 3.1. The Glycosylation Level of the Bacterial Protein rBCG-Rv1002c Is Significantly Increased

**Figure 1:**
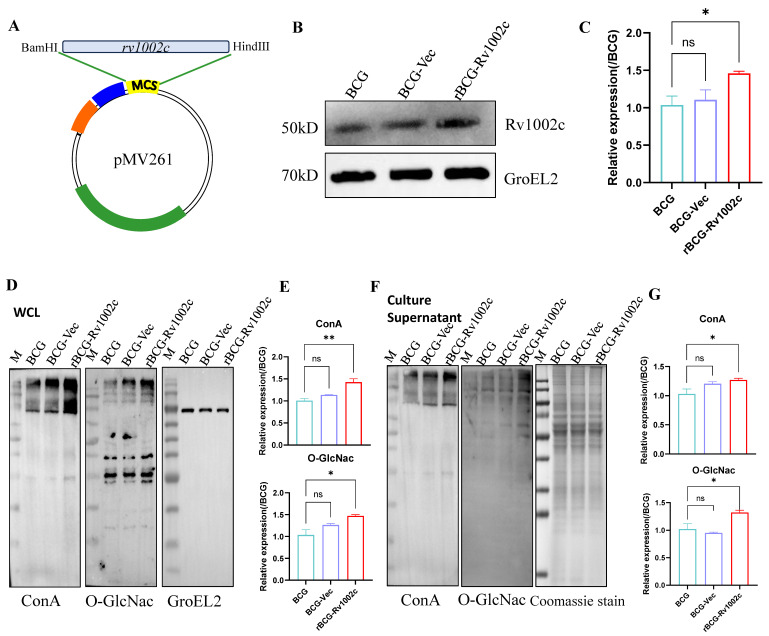
Construction and validation of rBCG-1002c. (**A**) Schematic diagram of pMV261-Rv1002c plasmid construction. (**B**) WB verified the expression level of Rv1002c. (**C**) The bands in (**B**) were analyzed semi-quantitatively and statistically using ImageJ version 1.8.0. The calculations were performed by obtaining the semi-quantitative ratio of the target gene to the internal reference for each group and then calculating the statistical expression relative to the BCG group, using the BCG group as a baseline. WB verified the glycoprotein expression levels of the different strains using ConA and O-GlcNac to detect glycoprotein content in whole cell lysates (**D**) and culture supernatants (**F**), respectively. (**E**) The bands in (**D**) were analyzed semi-quantitatively and statistically using ImageJ. (**G**) The bands in (**F**) were analyzed semi-quantitatively and statistically using ImageJ. ns, No significance; *, *p* < 0.05, **, *p* < 0.01.

The pMV261 plasmid was used to express Rv1002c in BCG strains, and a control of BCG-Vec was constructed (Figure 1A). After WB validation, rBCG-Rv1002c effectively overexpressed the Rv1002c protein compared to the BCG and empty groups (Figure 1B,C). Considering that Rv1002c serves as an O-glycosyltransferase in *Mycobacterium*, we detected the glycosylation levels of whole cell lysate (WCL) and secreted supernatant proteins of BCG, BCG-Vec, and rBCG-Rv1002c to investigate whether the O-glycosylation level of BCG bacterial proteins changed after Rv1002c overexpression. The proteins in the WCL and culture supernatants of the three strains were examined using concanavalin A (ConA) and O-GlcNac antibodies. The glycosylation level of the protein of rBCG-Rv1002c was increased in both the WCL and culture supernatants (Figure 1D–G). We also performed mass spectrometry of O-glycosylation-modified proteins in the culture supernatants of the three strains. We found that the number of O-glycosylation-modified proteins secreted by rBCG-Rv1002c was significantly elevated (Supplementary Table S1). These results suggest that Rv1002c overexpression promoted elevated levels of O-glycosylation in BCG bacteriophages.

### 3.2. rBCG-Rv1002c Significantly Enhances the Immunomodulatory Function of Macrophages

**Figure 2:**
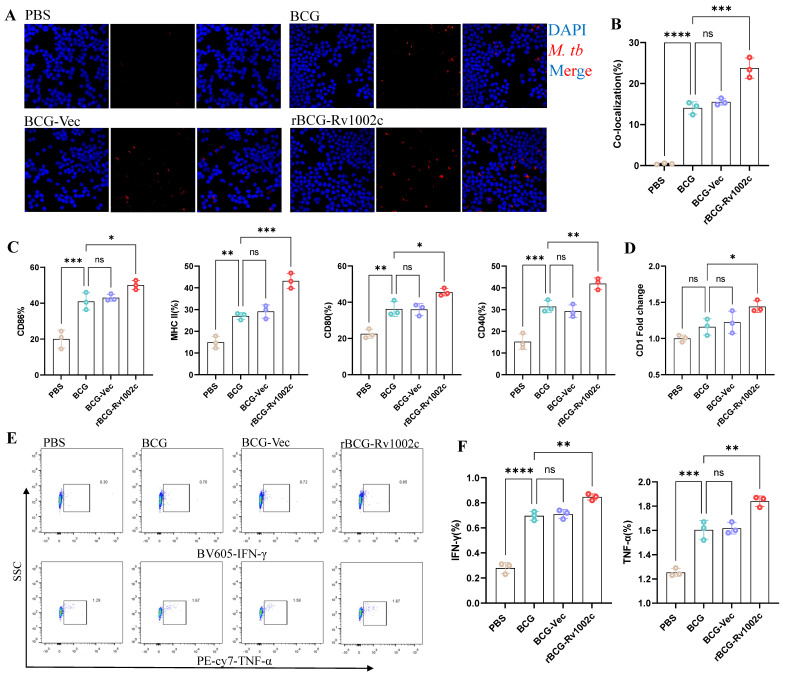
rBCG-Rv1002c enhances the immune function of macrophages. Detection of cell samples at specific time points after infestation of THP-1 macrophages with the different strains. (**A**) At 24 h post-infection, heat-inactivated *M. tb-mCherry* was added, incubated for 12 h, and washed three times with PBS before the phagocytosis of bacteria by macrophages was detected using immunofluorescence. Original magnification, 63×. (**B**) Statistical results for macrophage phagocytosis. (**C**) Expression of surface markers, including CD86, MHC-II, CD80, and CD40, using a flow-through assay after 24 infections with different strains. (**D**) Macrophage CD1 expression levels after 24 h of infection with the different strains. (**E**,**F**) After infection with different strains, mixed lymphoid cultures were performed, and the secretion levels of TNF-α and IFN-γ in Jurkat cells were detected using a flow assay. Data are shown as means ± SD, *n* = 3. Statistical analysis was performed using one-way ANOVA (ns, No significance; *, *p* < 0.05, **, *p* < 0.01, ***, *p* < 0.001, ****, *p* < 0.0001).

O-glycosylation modification is an important pathway for *Mycobacterium* to successfully interact with the host–pathogen and establish infection. Variations in the quantities of immunogenic glycosylated proteins in strains can directly impact the host’s capacity to identify pathogens and react to infection. We tested the immune function of THP1-driven macrophages after infection to investigate the effect of rBCG-Rv1002c on host immune function. After 24 h of infection with different strains, heat-inactivated *M. tb* was added to the culture system at a cell: *M. tb* = 1:5. After 12 h, the phagocytic ability of macrophages was assessed using immunofluorescence. The results showed that macrophages infected with rBCG-Rv1002c had a stronger phagocytic ability against *M. tb* than the BCG and plasmid control (Figure 2A,B). This also provides a basis for the enhanced antigen presentation by macrophages. rBCG-Rv1002c infection significantly upregulated immune regulatory molecules on the macrophage surface, including CD40, CD80, CD86, and MHC-II (Figure 2C). Additionally, the host showed significantly upregulated CD1 expression, which was primarily dependent on *M. tb* glycolipid antigen recognition (Figure 2D). We performed a mixed lymphoid assay with Jurkat cells and a flow assay of T cells to further investigate the effect of rBCG-Rv1002c on macrophage antigen presentation. We found that rBCG-Rv1002c infection of macrophages activated the T cell response, prompting T cells to significantly secrete IFN-γ and TNF-α (Figure 2E,F and Figure S2). These results indicate that rBCG-Rv1002c can significantly enhance the phagocytosis and presentation functions of macrophages.

### 3.3. rBCG-Rv1002c Enhances the Immune Response of Macrophages via the NF-κB Pathway

**Figure 3:**
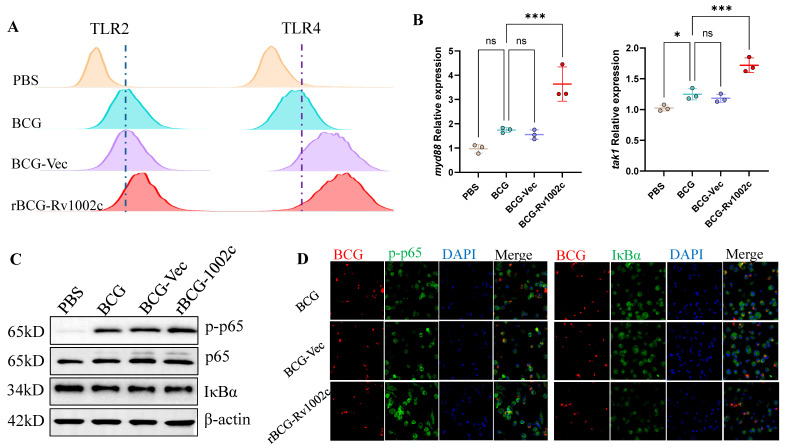
rBCG-Rv1002c activates the NF-κB pathway in macrophages. (**A**) TLR2 and TLR4 expression levels in macrophages after bacterial infection. At 48 h after bacterial infection, cells were harvested and stained with anti-TLR2-FITC and anti-TLR4-PE antibodies, washed twice with PBS after 20 min, and analyzed using a BD Fortessa flow cytometer to count the mean fluorescence intensity (MFI) of the different groups for analysis. (**B**) The myD88 and tak1 expression was detected using qPCR 48 h after bacterial infection. Activation of the NF-κB pathway was detected 48 h after bacterial infection using (**C**) WB and (**D**) immunofluorescence to detect activation of the NF-κB pathway 48 h after bacterial infection. Original magnification, 63×. Data are presented as means ± SD, *n* = 3. Statistical analysis was performed using one-way ANOVA (ns, No significance; *, *p* < 0.05, ***, *p* < 0.001).

We examined macrophage pattern recognition receptors after infection to investigate how rBCG-Rv1002 modulated the immune response of THP1-driven macrophages. We found that TLR2 and TLR4 receptor expression levels were significantly upregulated in macrophages (Figure 3A), while the expression of downstream molecules such as *myD88* and *tak1* was also upregulated (Figure 3B). The *myD88* is an important molecule for recognizing *M. tb* in macrophages. Phagocytosis in *M. tb* is activated by this recognition, effectively activating the NF-κB pathway. Detection of functional proteins of the NF-κB pathway 6 h and 48 h after infection of macrophages with rBCG-Rv1002c revealed that rBCG-Rv1002c significantly promoted p65 phosphorylation and inhibited IκBα expression (Figure 3C,D and Figure S3). RNA-Seq of lungs from mice immunized with different strains for four weeks revealed significant activation of TLR receptors and the NF-κB pathway in rBCG-Rv1002c-immunized mice (Figure S4). This result indicated that rBCG-Rv1002 promoted the NF-κB pathway in macrophages and enhanced the immune response of macrophages.

### 3.4. rBCG-Rv1002c Enhances the Response of the Innate Immune System in Mice

**Figure 4:**
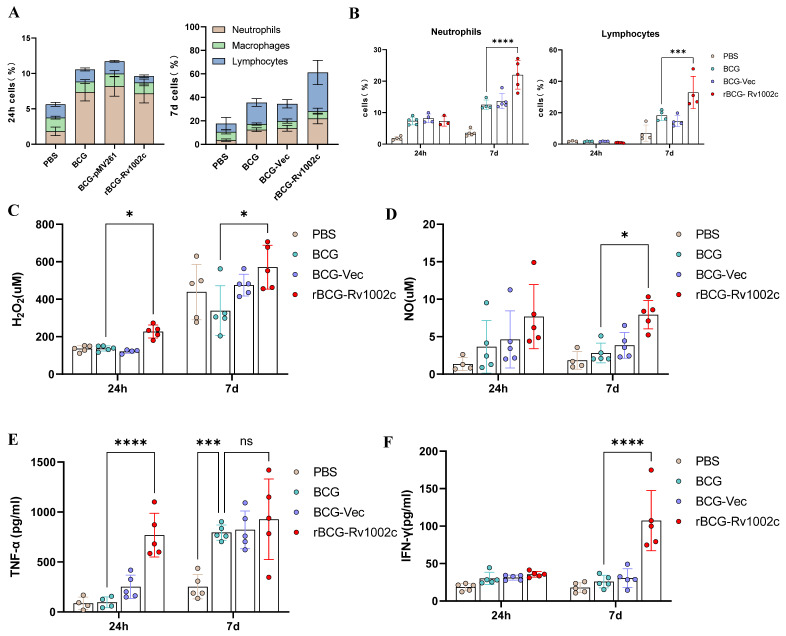
rBCG-Rv1002c promotes strong activation of intrinsic immune cells in mice. (**A**,**B**) Proportions of lymphocytes, neutrophils, and macrophages in the peritoneal cavity of mice at 24 h and seven days after immunization. The peritoneal immune cells were removed, and after stimulation with antigens in vitro, the secretion of the corresponding factors was assessed. (**C**) H_2_O_2_ secretion levels in mouse peritoneal cells after immunization with different strains. (**D**) NO secretion levels in mouse peritoneal cells after immunization with different strains. TNF-α (**E**) and IFN-γ (**F**) secretion levels in mouse peritoneal cells after immunization with different strains. Data are presented as means ± SD, *n* = 5. Statistical analysis was performed using two-way ANOVA (ns, No significance; *, *p* < 0.05, ***, *p* < 0.001, ****, *p* < 0.0001).

We immunized mice and evaluated the impact of various strains on the induction of innate immune cells to determine whether rBCG-Rv1002c was more immunogenic than BCG. The distribution of peritoneal cell populations was analyzed using flow cytometry within a short period after immunization, specifically the proportion of lymphocytes (CD45^+^CD3^+^), neutrophils (CD45^+^CD11b^+^Ly6C^+^Ly6G^+^), and macrophages (CD45^+^CD11b^+^F4/80^+^) (Figure S5). At 24 h after immunization, the number of neutrophils in the peritoneal cells of mice was significantly higher in the BCG- and rBCG-Rv1002c groups, without significant difference between the groups. Seven days after immunization, the total number of neutrophils and lymphocytes in the peritoneal cells of mice increased in the BCG and rBCG-Rv1002c groups (Figure 4A). The proportion of neutrophils and lymphocytes was significantly higher in the rBCG-Rv1002c-immunized group than in the BCG group (Figure 4B). This suggests that rBCG-Rv1002 can effectively stimulate neutrophil proliferation and T lymphocyte recruitment. Activated macrophages and neutrophils produce NO and H_2_O_2_ during oxidative burst, which can kill *M. tb* and is an important defense mechanism against *M. tb* invasion. After stimulating mouse peritoneal cells with the antigen Rv1002c for 12 h, the cell culture supernatants were collected, and the NO and H_2_O_2_ secretion levels in the supernatants were examined. The rBCG-Rv1002 group induced a higher level of H_2_O_2_ secretion compared to the BCG and BCG-Vec groups after 24 h and seven days of immunization (Figure 4C). The level of NO secretion was significantly enhanced seven days after immunization, whereas the rBCG-Rv1002 group did not significantly increase 24 h after immunization (Figure 4D). We also examined cytokine secretion after stimulation with the antigen Rv1002c. After 24 h of immunization, the rBCG-Rv1002c group induced a higher TNF-α secretion level than the BCG group. Seven days after immunization, the rBCG-Rv1002c group exhibited no significant change in TNF-α secretion level but significantly higher IFN-γ secretion (Figure 4E,F). These results suggest that rBCG-Rv1002c can recruit more innate immune cells and activate them to secrete large amounts of bactericidal substances and inflammatory cytokines, which are important for activating adaptive immunity.

### 3.5. rBCG-Rv1002c Activates Adaptive Immunity and Provides Long-Lasting Protection in Mice

**Figure 5:**
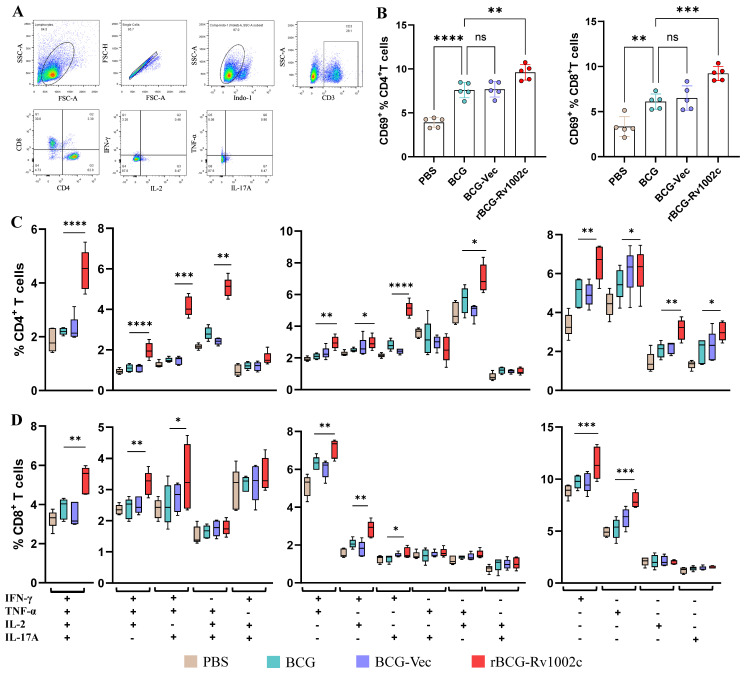
rBCG-Rv1002c significantly enhances the adaptive immune response. Four weeks after immunization with the different strains, the mice were sacrificed, and lymphocytes from the spleen were examined. (**A**) Logical schematic of the T cell assay. (**B**) The degree of activation of CD4^+^ and CD8^+^ was assayed. (**C**,**D**) Proportion of different subpopulations of CD4^+^ and CD8^+^ T cells. Data are presented as means ± SD, *n* = 5. Statistical analysis was performed using one-way ANOVA (ns, No significance; *, *p* < 0.05, **, *p* < 0.01, ***, *p* < 0.001, ****, *p* < 0.0001).

We examined mice four weeks after immunization to study the adaptive immune response in different experimental groups. This study found that the potency of specific IgG and IgA of BCG WCL in the serum of mice was significantly higher in the rBCG-Rv1002c group than in the BCG group (Figure S6A,B). T cells were isolated from the spleens of immunized and control mice, and the degree of T cell activation was assessed. The rBCG-Rv1002c group mice displayed greater T cell activation (CD69^+^) for CD4^+^ T and CD8^+^ T (Figure 5A,B). Multifunctional T cells expressing a variety of cytokines (IFN-γ, TNF-α, IL-17, and IL-2) are associated with host protection against infectious diseases [29]. Additionally, the presence of these multifunctional T cells is important for effective vaccines against intracellular bacterial pathogens [30]. Therefore, we analyzed multifunctional cells in different T cell subsets in the spleen. Interestingly, rBCG-Rv1002c immunization significantly increased the frequency of CD4^+^ and CD8^+^ T cells expressing single, double, triple, and quadruple positives compared to the other groups. Particularly, the rBCG-Rv1002c immune group showed a significantly increased frequency of multifunctional CD4^+^ and CD8^+^ T cells compared to the other groups (Figure 5C). In conclusion, we observed that rBCG-Rv1002c immunization greatly enhanced antigen-specific Th1 and Th17 responses, as well as the pro-inflammatory cytokines TNF-α and IL-2, all of which have been well documented to confer protection against TB. Furthermore, rBCG-Rv1002c immunization significantly promoted CD107a and GrzB expression in CD8^+^ T cells (Figure S6C,D), contributing to the CTL effect. We also assessed the immune memory of the T cells. The rBCG-Rv1002c immunization promoted the generation of more T_CM_ (CD44^+^CD62L^+^) and T_EM_ (CD44^+^CD62L^−^) cells in the mouse splenocytes (Figure S6E), suggesting that the vaccine may provide long-term immune protection.

### 3.6. rBCG-Rv1002c Provides Greater Protection against M. tb Infection than BCG

**Figure 6:**
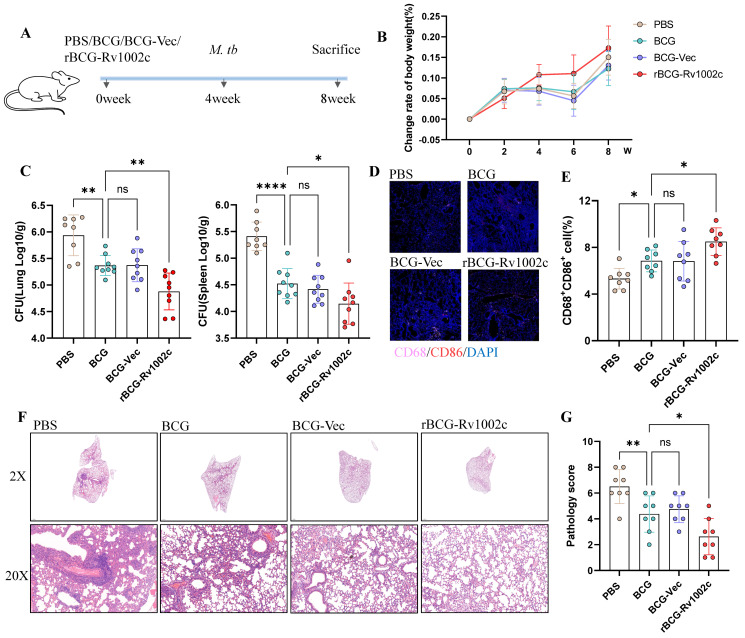
rBCG-Rv1002c showed better immune protection against *M. tb*. (**A**) Schematic representation of the experimental plan for mouse infection. (**B**) Body weight changes in the mice after bacterial infection. (**C**) CFU counts in the lungs and spleen after bacterial infection. (**D**,**E**) Immunofluorescence images of macrophage typing in mouse lungs 28 days after bacterial infection, represented in the field of view, with an analysis of the proportion of CD86-positive macrophages. (**F**) H&E staining of the lungs following bacterial infection. (**G**) The degree of lesions in the lungs was scored statistically based on H&E staining results. Data are presented as means ± SD, *n* = 8. Statistical analysis was performed using one-way ANOVA (ns, No significance; *, *p* < 0.05, **, *p* < 0.01, ****, *p* < 0.0001). Original magnification, 25× (**D**).

We infected mice with H37Rv 4W after immunization to investigate whether rBCG-Rv1002c had a stronger immunoprotective effect (Figure 6A). After *M. tb* infection, we found that the rBCG-Rv1002c group of mice had a more pronounced increase in body weight (Figure 6B). Following four weeks of infection, the mice’s lungs and spleens had their bacterial loads measured. However, the CFU levels were much lower in the lungs and spleen of the rBCG-Rv1002c group than in the BCG group, indicating that the immunological group could effectively reduce the bacterial burden compared to the PBS group (Figure 6C). The rBCG-Rv1002c group was better able to activate lung macrophages and convert them to the M1 phenotype, contributing to host clearance of bacteria (Figure 6D,E). Furthermore, the rBCG-Rv1002c group’s lungs had far fewer pathological alterations, including fewer parenchymal lesions and a clearer alveolar structure, than the PBS and BCG groups (Figure 6F). Additionally, the pathological scores showed that rBCG-Rv1002c could considerably lessen lesions in the mice’s lungs caused by an *M. tb* infection (Figure 6G). This suggests that rBCG-Rv1002c promotes a higher protective effect against *M. tb* infection in the host than BCG.

## 4. Discussion

BCG is a live attenuated vaccine, and chemotherapy and BCG have been widely used to treat and prevent TB. However, studies have shown that previous exposure to *Mycobacterium* can inhibit the activity of BCG, resulting in inadequate protection in areas of high TB prevalence [31,32]. Significant progress has been made in TB vaccine development over the past decade. As first-generation TB vaccine candidates move into more advanced clinical trials, it is recognized that there is a need to diversify vaccines to maximize the chances of bringing improved TB vaccines into widespread use [14,33,34]. In this study, we demonstrated that rBCG-Rv1002c is an effective vaccine candidate.

Rv1002c is important for *M. tb* virulence and in vitro growth. This mannosyltransferase is more highly expressed in the most virulent Beijing strain than in other *M. tb* families, supporting its role in virulence [27,35]. Additionally, intracellular persistence and proliferation of Rv1002c mutants in mouse and human macrophages and virulence in SCID mice were impaired, suggesting that O-mannosylation is an important pathway for successful host–pathogen interactions and establishing infectivity by *M. tb* [27]. M.sm overexpressing Rv1002c also demonstrated better in vivo survival than the wild-type strain [28]. Reduced immunodominant glycosylated protein levels in ΔRv1002c strains, many of which are known TLR-2 agonists, may directly impact the host’s ability to recognize pathogens and respond appropriately to infection [36,37]. The rBCG-Rv1002c strain secretes more glycosylated proteins, promoting phagocytic processing and antigenic presentation by macrophages [21].

Following exposure to *M. tb*, approximately 70% of the population is not infected, highlighting the importance of early clearance by innate immunity [38]. The response of alveolar epithelial cells to *M. tb* infection results in the secretion of pro-inflammatory cytokines, such as TNF-α and IFN-γ, activating the autophagy–lysosomal pathway and clearing invasive *M. tb* from alveolar macrophages [39,40]. The rBCG-Rv1002c secreted more glycosylation-modified proteins, contributing more to the recognition and phagocytosis of antigens by macrophages. The rBCG-Rv1002c-infected macrophages upregulated the TLR and CD1 expression on the macrophage surface, activated the NF-κB pathway, and facilitated the release of large amounts of NO and H_2_O_2_, which could control bacterial growth more effectively. The phagocytic processing of antigens by macrophages also affected their ability to present antigens, and the expression of molecules such as CD40 and CD86 on the rBCG-Rv1002c-infected macrophage surface was significantly increased. Mice vaccinated with rBCG-Rv1002c produced more multifunctional specific CD4^+^ T cells, including Th17 cells co-expressing IFN-γ, TNF-α, and IL-2, key cytokines that contributed to the control of *M. tb*. CD4^+^ T cells are essential for the immunological control of *M. tb,* and T cell differentiation is thought to be a determinant of pulmonary protective factors [41]. Immunization with rBCG-Rv1002c was associated with sustained Th17 induction and memory CD4^+^ T cells, with superior protective capacity compared with BCG. CD8^+^ lymphocytes are important for limiting the establishment and spread of infection, as demonstrated in the NHP model [42,43]. The rBCG-Rv1002c-immunized mice exhibited a stronger CTL effect with an increased release of granzyme perforin. These improvements in T cell immunity contributed to greater protection against *M. tb* infection in rBCG-Rv1002c-immunized mice by controlling bacterial proliferation in vivo. Compared to the BCG-immunized group, the rBCG-Rv1002c-immunized mice could limit bacterial growth during *M. tb* infection, and their weight loss and lung lesions were better managed.

In conclusion, our results suggest that the rBCG-Rv1002c vaccine activated phagocytosis and presentation by macrophages, enhanced Th1/Th17 and CD8^+^ CTL immune responses, and increased memory T cell development. The rBCG-Rv1002c provided more significant immunoprotective effects than BCG, as demonstrated by significantly reduced lung bacterial load and histological damage. Our study suggests that rBCG-Rv1002c is a highly promising vaccine that could be better than BCG for preventing TB infection.

## Data Availability

The original contributions presented in this study are included in the article or Supplementary Materials. Further inquiries can be directed to the corresponding author.

## Supplementary Materials

The following supporting information can be downloaded at: https://www.mdpi.com/article/10.3390/vaccines12060622/s1, Figure S1: Rv1002c protein was successfully induced in *E. coli* BL21 by supplementation with 0.2 mM isopropyl β-D-thiogalactoside (IPTG) for 8 h. The lane selected in the red box is the purified Rv1002c protein. Figure S2: rBCG-Rv1002c activates the NF-κB pathway in macrophages. After infection with different strains, mixed lymphoid cultures were performed, and the mRNA levels of TNF-α and IFN-γ in Jurkat cells were detected using RT-QPCR. Data are shown as means ± SD, *n* = 3. Statistical analysis was performed using one-way ANOVA (ns, No significance; *, *p* < 0.05, **, *p* < 0.01). Figure S3: rBCG-Rv1002c activates the NF-κB pathway in macrophages. Activation of the NF-κB pathway was detected 6 h after bacterial infection using WB. Figure S4: KEGG analysis of lungs after immunization with different strains. RNA from mouse lungs was analyzed for KEGG after 4 weeks of immunization with rBCG-Rv1002 and BCG. Figure S5: Logical schematic of the cell assay. The distribution of cell populations of peritoneal cells was analyzed by flow cytometry within a short period of time after immunization, analyzing the proportions of lymphocytes (CD45^+^CD3^+^), neutrophils (CD45^+^CD11b^+^Ly6C^+^Ly6G^+^), and macrophages (CD45^+^CD11b^+^F4/80^+^). Figure S6: rBCG-Rv1002c significantly enhanced the adaptive immune response. Four weeks after immunization with the different strains, the mice were sacrificed and lymphocytes from the spleen were examined. (A) BCG WCL-specific IgG titers in serum. (B) BCG WCL-specific IgA titers in serum. (C,D) Expression of functional factors in CTL cells. (E) Proportion of memory T cells after immunization with different strains. Data are shown as means ± SD, *n* = 5. Statistical analysis was performed using one-way ANOVA (ns, No significance; *, *p* < 0.05, **, *p* < 0.01, ****, *p* < 0.0001). Table S1: Secretion level of O-glycosylation protein in culture filtrate.
