# Supplementary material for: Enhanced Glycosylation Caused by Overexpression of Rv1002c in a Recombinant BCG Promotes Immune Response and Protects against Mycobacterium tuberculosis Infection
Source: Vaccines (Basel). 2024 Jun 4;12(6):622. doi: 10.3390/vaccines12060622 (PMC11209282; doi:10.3390/vaccines12060622)
Supplement: Supplementary file 1 [file vaccines-12-00622-s001.zip › Supplementary figures.pdf]

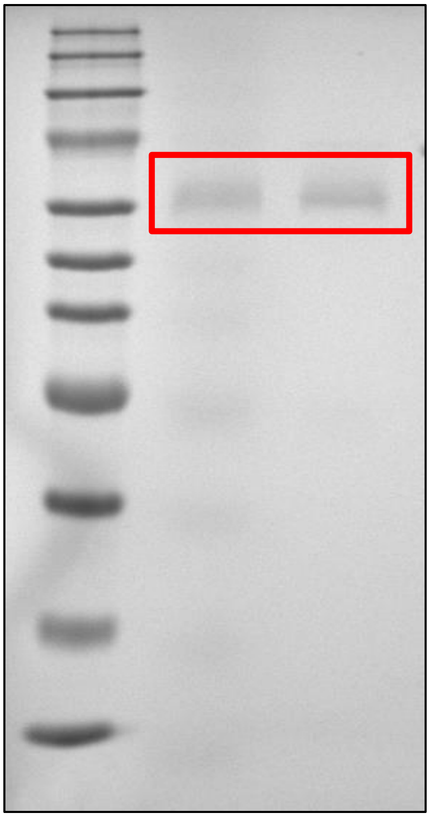

Figure S1. Rv1002c protein was successfully induced in *E. coli* BL21 by supplementation with 0.2 mM isopropyl β-D-thiogalactoside (IPTG) for 8 h. The lane selected in the red box is the purified Rv1002c protein.

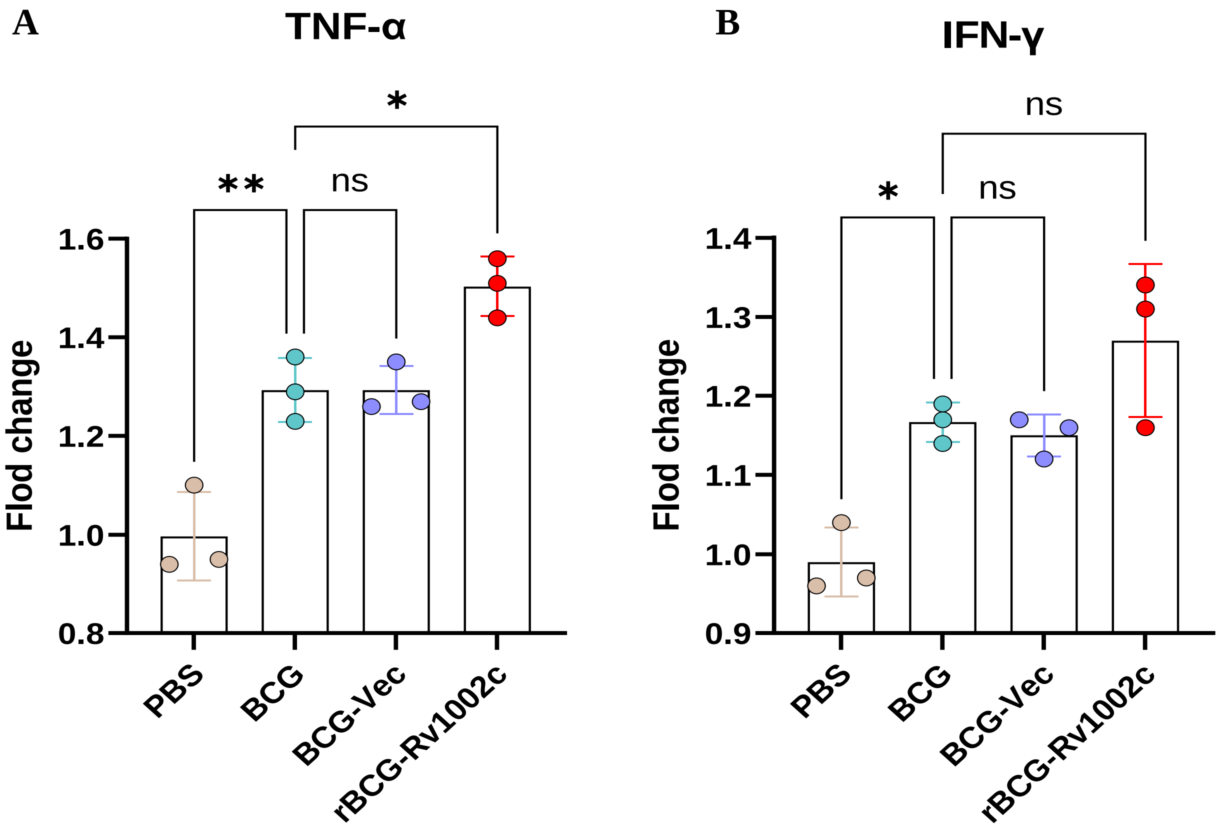

Figure S2. rBCG-Rv1002c activates the NF-κB pathway in macrophages. After infection with different strains, mixed lymphoid cultures were performed, and the mRNA levels of TNF-α and IFN-γ in Jurkat cells were detected using RT-QPCR. Data are shown as mean± SD, n = 3. Statistical analysis was performed using one-way ANOVA (ns, No significance; *, $P < 0.05$, **, $P < 0.01$).

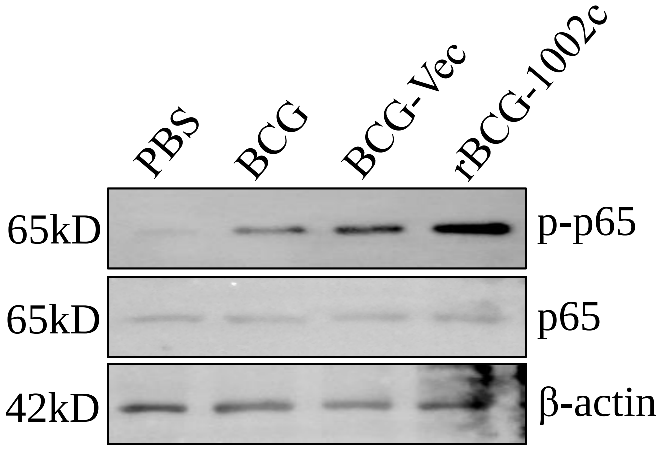

Figure S3. rBCG-Rv1002c activates the NF-κB pathway in macrophages. Activation of the NF-κB pathway was detected 6 h after bacterial infection using WB.

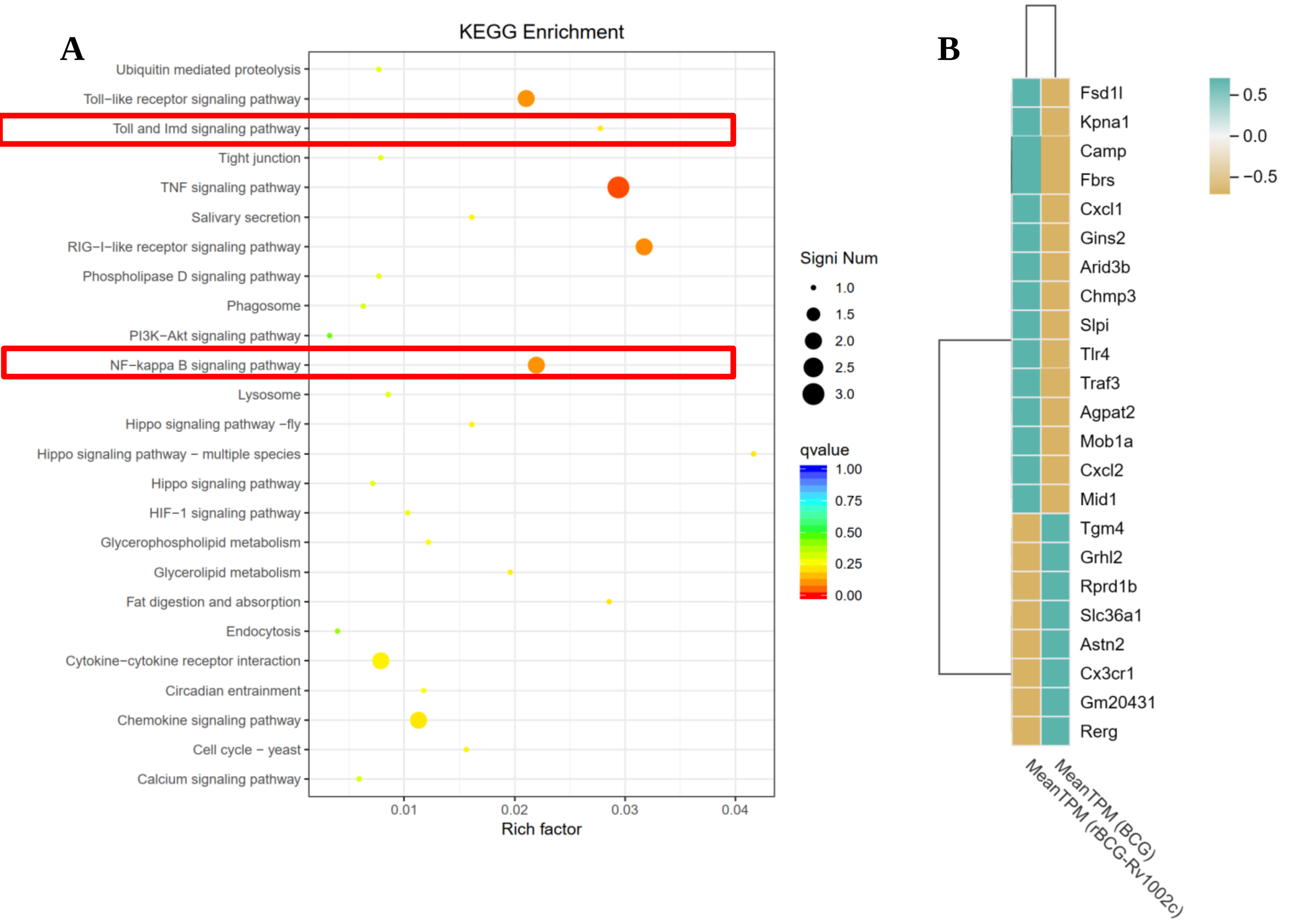

Figure S4 KEGG analysis of lungs after immunization with different strains. RNA from mouse lungs was analyzed for KEGG after 4 weeks of immunization with rBCG-Rv1002 and BCG.

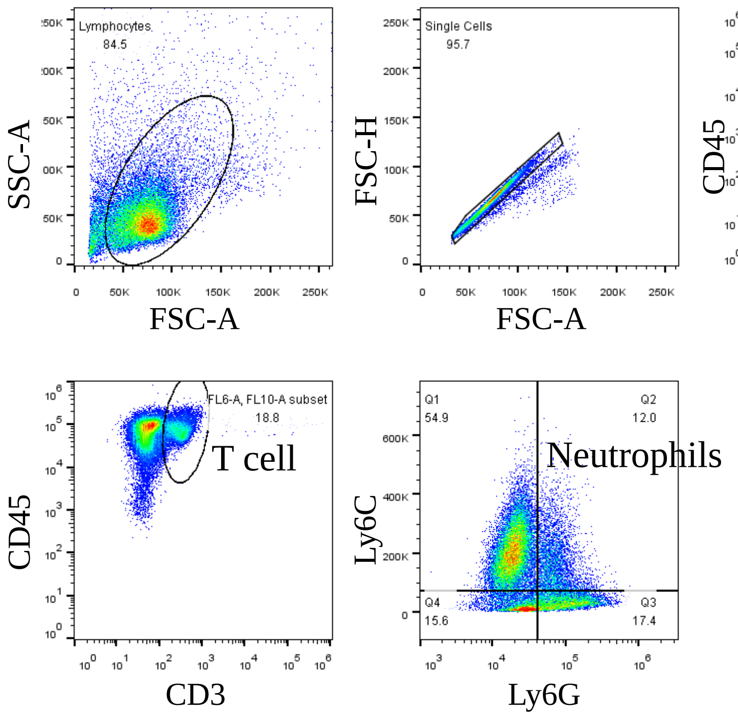

Figure S5. Logical schematic of the cell assay. The distribution of cell populations of peritoneal cells was analyzed by flow cytometry within a short period of time after immunization, analyzing the proportions of Lymphocytes ($CD45^+CD3^+$), Neutrophils ($CD45^+CD11b^+Ly6C^+Ly6G^+$), and Macrophages ($CD45^+CD11b^+F4/80^+$).

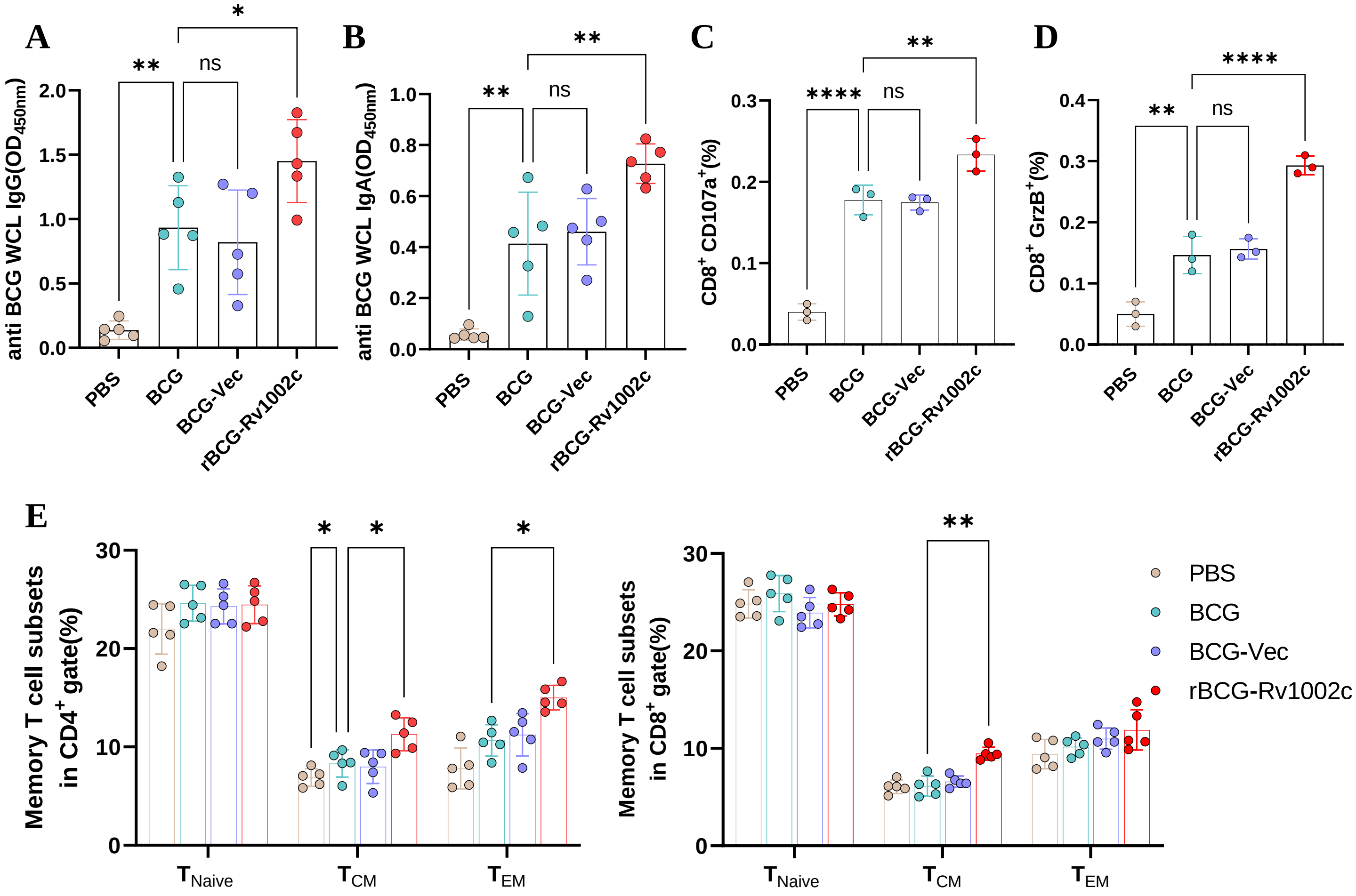

Figure S6. rBCG-Rv1002c significantly enhanced the adaptive immune response. Four weeks after immunization with the different strains, the mice were sacrificed and lymphocytes from the spleen were examined. (**A**) BCG WCL-specific IgG titers in serum. (**B**) BCG WCL-specific IgA titers in serum. (**C,D**) Expression of functional factors in CTL cells. (**E**) Proportion of memory T cells after immunization with different strains. . Data are shown as mean± SD, n = 5. Statistical analysis was performed using one-way ANOVA (ns, No significance; *, P < 0.05, **, P < 0.01, ****, P < 0.0001).
