# Supplementary material for: Enhanced Glycosylation Caused by Overexpression of Rv1002c in a Recombinant BCG Promotes Immune Response and Protects against Mycobacterium tuberculosis Infection
Source: Vaccines (Basel). 2024 Jun 4;12(6):622. doi: 10.3390/vaccines12060622 (PMC11209282; doi:10.3390/vaccines12060622)
Supplement: Supplementary file 1 [file vaccines-12-00622-s001.zip › Supplementary Table S1 Secretion level of O-glycosylation protein in culture filtrate.pdf]

Supplementary Table S1: Secretion level of O-glycosylation protein in culture filtrate.

Uniprot	Name	Description	=BCG-Vec/BCG	=rBCG-Rv1002c/BCG
P9WIR7	Apa(Rv1860)	Glycoprotein, secreted antigen; Adhesin	0.747710443	2.889586256
P9WK55	LprA(Rv1270c)	TLR2 agonist	0.904585756	2.427715405
P9WNF3	MPT83(Rv2873)	Latent antigen	0.594703937	2.482051177
P9WGU1	PstS-1(Rv0934)	Glycoprotein, B-cell antigen	0.92764477	3.591215325
P9WQP3	FbpA(Rv3804c)	Diacylglycerol acyltransferase	1.318563492	3.261486392
P9WK65	Lppx(Rv2945c)	Lipoprotein	0.868654456	2.635984096
P9WGT7	PstS-3(Rv0928)	Phosphate binding protein, secreted antigen	0.970002504	2.743656129
P9WGV3	achY(Rv3248c)	Adenosine homocysteine enzyme	0.829491343	1.517866987
O50430	TB8.4(Rv1174c)	T-cell antigen	1.318563492	4.752471844
P9WK45	LprG(Rv1411c)	Mediating the expression of LAM	0.795624311	2.810953427
O05842	Rv3224	Short chain dehydrogenase or reductase	0.695616254	2.740235582
P9WK61	LpqH(Rv3763)	Glycoprotein adhesins, and virulence related	0.882437493	2.740235582
O53572	Rv3587c	Unknown membrane protein	0.976808691	3.452064709

Note: The data in the table are calculated as the protein amount in BCG-Vec and rBCG-Rv1002c using the protein amount in the BCG group as the baseline. BCG protein content is the mass spectrometry peak area value.
